# Three-year trends in dietary behaviours among mothers, teenagers and children from SNAP-Ed (Supplemental Nutrition Assistance Program–Education) eligible households across California

**DOI:** 10.1017/S1368980019003197

**Published:** 2019-11-20

**Authors:** Fred Molitor, Celeste Doerr, John Pugliese, Lauren Whetstone

**Affiliations:** 1 California State University, Sacramento, College of Continuing Education and Department of Communication Studies, 6000 J Street, Sacramento, CA 95819, USA; 2 Public Health Institute Center for Wellness and Nutrition, Sacramento, CA, USA; 3 California Department of Public Health, Sacramento, CA, USA

**Keywords:** Behaviour surveillance, Supplemental Nutrition Assistance Program–Education, Population-based survey, Low-income population, Fruit and vegetable consumption, Sugar-sweetened beverages, Diet quality

## Abstract

**Objective:**

To examine trends from 2015 to 2017 in dietary behaviours and diet quality among low-income mothers, teenagers and children.

**Design:**

Cross-sectional telephone surveys using a validated 24 h dietary assessment.

**Setting:**

Randomly sampled households with incomes ≤185 % of the US federal poverty level across California.

**Participants:**

Survey participants were 13 247 mothers (≥18 years), 3293 teenagers (12–17 years) and 6043 children (5–11 years). Respondents were mostly Latino.

**Results:**

Over the 3-year study period, consumption of fruits and vegetables with and without 100 % fruit juice increased (*P* ≤ 0·05) by at least 0·3 cups/d for mothers, teenagers and children. Intake of water also increased (*P* ≤ 0·001) by more than 1 cup/d for mothers and children and 2 cups/d for teenagers. Sugar-sweetened beverage (SSB) consumption was unchanged over the 3 years. Overall diet quality, as assessed by the Healthy Eating Index-2015, improved (*P* ≤ 0·01) for mothers, teenagers and children. Covariates for the fifteen regression models (three age groups by five outcome variables) included race/ethnicity, age, education for mothers, and gender for teenagers and children.

**Conclusions:**

The observed increases in fruit and vegetable intake and improvements in overall diet quality during the 3-year period suggest that low-income Californians may have lowered their risk of preventable diseases. However, more intense or strategic SSB-reduction interventions are required. Regional- or state-level, population-based surveillance of dietary behaviours is useful for public health nutrition policy and programme decision making, and can be used to assess potential trends in future negative health outcomes and related costs associated with poor dietary behaviours within at-risk populations.

Improving the quality of dietary intake has the potential to reduce disease rates in a population. Healthier dietary behaviours can decrease the burden on individuals and society to treat illnesses and protect individuals from the psychological and physical suffering that accompanies preventable diseases.

Fruit and vegetable intake is a core indicator of healthy dietary behaviours^(1)^. Low consumption of fruits and vegetables is a primary behavioural risk factor for preventable cancers in adults in the USA^(2)^. In fact, 15 940 cancer deaths in 2014 were attributed to diets low in fruits and vegetables^(2)^. Researchers have also concluded that eating fruits and vegetables decreases the risk of CVD mortality^(3)^. Specifically, the synthesis of findings from several published studies led to the conclusion that the chance of dying from a CVD is reduced by 4 % for each serving of fruits and vegetables eaten daily^(3)^. Eating more fruits and vegetables has also been associated with a reduced risk of type 2 diabetes^(4)^.

In line with the 2015–2020 Dietary Guidelines for Americans^(5)^, consumption of sugar-sweetened beverages (SSB) is also identified as a key indicator of a healthy diet^(1)^. Whereas increased fruit and vegetable intake appears to be protective against the most common, costly and preventable diseases, consumption of SSB has contrary effects. SSB intake is associated with an increased risk of metabolic syndrome^(6,7)^ and type 2 diabetes and CVD independent of obesity^(8)^. In the context of drinking water instead of SSB, average cups of water consumed daily is a third important indicator of healthy dietary behaviours^(1)^.

Compared with higher-income persons, those eligible for the Supplemental Nutrition Assistance Program–Education (SNAP-Ed), defined as individuals with household incomes at or below 185 % of the US federal poverty level^(9)^, report lower levels of fruit and vegetable and higher levels of SSB intake^(10)^. Similarly, a systematic review of SSB consumption found that lower parental socio-economic status was strongly associated with higher SSB intake among young children^(11)^.

Approximately 12·8 million persons in California are SNAP-Ed eligible, representing 33·1 % of the State’s population in 2017^(12)^. SNAP-Ed interventions directed at this population are guided by Goal 1 of California’s SNAP-Ed programme state-wide goals and objectives: increase the consumption of fruits and vegetables, decrease the consumption of added sugar from beverages, and improve the dietary quality of meals and snacks among the SNAP-Ed eligible population^(13)^. California SNAP-Ed messages promote the consumption of water as an alternative to drinking SSB.

Dietary surveillance data available at the national level, such as from the National Health and Nutrition Examination Survey (NHANES) and the Behavioral Risk Factor Surveillance System (BRFSS), are of limited use when developing regional public health nutrition policies or interventions. This is due to the lag in the reporting of findings from ongoing surveillance surveys and the presentation of findings only at the national level. Moreover, trends in dietary outcomes at the national level cannot be used to adequately assess the effectiveness of regional interventions.

The California Family Health Study was developed to provide comprehensive, valid dietary intake data to track behavioural outcomes among the California SNAP-Ed eligible population for programme and evaluation purposes. A validated 24 h dietary recall assessment is administered annually by telephone to mothers, teenagers and children from randomly sampled SNAP-Ed eligible households. Mothers and their children are selected for interviews because the guiding principle of SNAP-Ed has long been that the programme has the greatest potential to improve the dietary behaviours of low-income households when it focuses on women, as nutritional gatekeepers of the family, and on children^(14)^. Dietary recall responses obtained from the California Family Health Study are converted to standardized quantities such as cups for fruits, vegetables, 100 % fruit juice, water and SSB. The detailed information on healthful and unhealthful dietary behaviours is also used to calculate measures of overall diet quality.

The objectives of the present study were to examine trends from 2015 to 2017 in the dietary behaviours and quality of low-income mothers, teenagers and children. The study results have the potential to inform public health nutrition policy and programme decision making, and to illuminate potential trends in future negative health outcomes and related costs associated with poor dietary behaviours within an at-risk population.

## Methods

The recruitment and interview procedures were implemented by the California State University, Sacramento, Public Health Survey Research Program during each of the 3 years of this study.

### Sampling

The sampling frames were created prior to the 2015, 2016 and 2017 surveys from the California Department of Health Services’ Medi-Cal (Medicaid in California) Eligibility Data System (MEDS) database. Households were included in the sampling frames if: (i) one or more members were eligible to receive the SNAP benefit in at least one of the previous 12 months; and (ii) the household contained at least one adult female and one child 5 to 17 years of age. Households were selected at random using custom programming written for the statistical software package SAS version 9.4. In 2015, households were randomly sampled within the seventeen largest California counties; households from an additional thirteen counties were sampled for the 2016 and 2017 surveys. In cases where a household had more than one young person, one teenager (12 to 17 years) or child (5 to 11 years) was selected at random using custom-programmed Sawtooth WinCati computer-assisted telephone interviewing software, version 6.0 (Sawtooth Technologies, Northbrook, IL, USA). Specifically, mothers were asked the ages of the children in the household. Interviewers entered each child’s age into the program and pseudorandom algorithmic assignment was used to identify the one teenager or child for the interview.

### Recruitment

Potential survey respondents were recruited using the names, addresses, telephone numbers, preferred languages, gender and ages of family members from the selected households from the MEDS database. This information was used to determine the mailing address and language (English or Spanish) for the letter briefly describing the study that was initially sent to sampled households. Bilingual staff followed-up with telephone calls to each household and read scripted voice mail messages when necessary. When telephone contact was established, interview staff confirmed household eligibility and the identity of the mother of children aged 5 to 17 years living in the household. For simplicity, we have termed these adult female household members ‘mothers’, although some may have been other primary caregivers. Eligible mothers were presented with a brief overview of the study’s purpose and procedures; they were also informed that they and their child would receive a $US 10 gift card for survey participation. The participating households were sent a study welcome packet containing a pictorial food and beverage portion-size booklet and measuring cups and spoons. A data collection telephone interview was scheduled at the end of the recruitment call. Gift cards were mailed to respondents following the interviews.

### Instruments

The 24 h dietary recall interviews were conducted in English or Spanish using the National Cancer Institute’s web-based Automated Self-administered 24-hour Dietary Assessment Tool (ASA24)^(15)^. Originally developed for online self-administration, the ASA24 protocols were modified through a collaboration with its developer, Westat, to accommodate telephone administration. Thus, in the present study, the ASA24 functioned as a web-based interviewer prompt as well as a data entry system. The adapted ASA24 administration protocols and standardized interviewer training procedures were applied to all 3 years of data collection. The ASA24 does not assess respondent demographics; responses to standardized questions regarding race/ethnicity, age, mother’s level of education, and gender for teenagers and children were recorded into a computer-assisted telephone interviewing system.

### Data collection

Interview staff asked mothers detailed questions about all the types of foods and beverages they had consumed over the previous 24 h. The ASA24 multiple-pass process involves first asking respondents to identify all meals and snacks, and then all foods and drinks consumed for each meal and snack over the last 24 h. The ASA24 offers detailed response options for general food categories. For example, if a respondent stated that she had ‘soup’ for lunch, the system generates a list of 197 types of soups (e.g. chicken with rice, cream of leek), which the interviewers used to prompt mothers to identify the specific type of soup consumed. Probes were used to assess additional details such as condiments added to all meal and snack items. The quantity and size of each identified item were assessed by asking mothers to refer to the study-supplied portion-size booklet or measuring cups and spoons. The portion-size booklet contained images of foods such as pizza slices and fruits of various sizes with descriptors such as ‘small’, ‘medium’, ‘large’ and ‘extra large’. Interviewers were trained to encourage mothers to utilize the images for comparable foods not included in the booklet, such as reporting the size of a tortilla using the pictures of pancakes. Bottles, cans, glasses, cups and mugs of varying sizes and corresponding fluid ounces were referenced for beverages, as depicted in the guide. Mothers were asked to refer to the measuring cups and spoons for cases in which the volume of the food or beverage item was not easily visualized. Once the portion size was identified, interviewers selected the corresponding response option using the web-based ASA24 system.

With the mother’s permission, the same interview procedures were employed with the teenager or child in the household. In households with children 6 to 11 years of age, the mother participated in the child interview (both the mother and child were on the telephone at the same time with the interviewer). This allowed the mother and child to discuss and confirm agreed-upon answers before the child (for 9- to 11-year-olds) or mother (for 6- to 8-year-olds) provided the answer that was recorded by the interviewers. For households with children aged 5 years, the mother participated in two interviews, one for herself, followed by one for her child. The average time for mothers’ interviews was 82 min and for teenagers’ or children’s interviews was 85 min.

### Outcome variables

Five outcome variables were examined in the present study: (i) cups of fruits and vegetables with 100 % fruit juice; (ii) cups of fruits and vegetables without 100 % fruit juice; (iii) cups of SSB; (iv) cups of water; and (v) Healthy Eating Index (HEI)-2015 scores. In line with California’s SNAP-Ed Goal 1, fruit and vegetable intake and SSB consumption were selected based on their known protective effects against increased risks for preventable diseases. Water was selected to assess the potential effectiveness of SNAP-Ed messages encouraging low-income Californians to replace SSB intake with water. HEI-2015 scores were selected to assess potential trends in overall diet quality above the intake of specific food and beverage types. These variables were either computed by the ASA24 system or were developed based on the response data retrieved from the system after each survey cycle. All five outcome variables were analysed as continuous variables.

#### Cups of fruits and vegetables with 100 % fruit juice

Cups of fruits and vegetables with 100 % fruit juice were derived from the sum of the ASA24-computed variables F_TOTAL (total intact fruits, whole or cut), V_TOTAL (total dark green, red and orange, starchy and other vegetables; excluding legumes) and F_JUICE (fruit juices, citrus and non-citrus). Including 100 % fruit juice with whole fruits and vegetables is in accordance with the US Department of Agriculture’s (USDA’s) SNAP-Ed Evaluation Framework that calls for including 100 % fruit juice when assessing fruit and vegetable consumption (indicator R2 under ‘Population Results’)^(1)^.

#### Cups of fruits and vegetables without 100 % fruit juice

For reasons including its high sugar content and low levels of fibre^(16)^, and the potential link with type 2 diabetes^(17)^, many public health professionals recommend eliminating or limiting fruit juice in children’s^(16)^ and families’^(18)^ diets, especially those from low-income populations. However, the conclusion of experts participating in a roundtable discussion was that there is no science-based reason to restrict access to 100 % fruit juice in public health nutrition policy and programmes, and that reducing or eliminating 100 % fruit juice could lead to unintended consequences^(19)^. Yet, recent evidence suggests that reducing the availability of fruit juice may lead to increased consumption of fruit and milk^(20)^. To ensure that any changes observed for measures of fruit and vegetable consumption were not overly influenced by intake of 100 % fruit juice, and to provide population-based consumption estimates for comparisons with other studies excluding fruit juice, we examined cups of fruits and vegetables excluding 100 % fruit juice: sum of ASA24 variables F_TOTAL and V_TOTAL only.

#### Cups of sugar-sweetened beverages

SSB intake was calculated as total cups of sugar-sweetened soda, energy (e.g. Red Bull®), fruit (e.g. Sunny Delight®), sports (e.g. Gatorade®) drinks, and coffee or tea beverages. Excluded were beverages with artificial sweeteners or ‘diet’ soda.

#### Cups of water

Cups of water were derived from reported intake of tap or unsweetened bottled water, including flavoured or vitamin-fortified water.

#### Health Eating Index-2015 scores

Diet quality was assessed by HEI-2015 composite scores. HEI-2015 scores range from 0 to 100, with higher values aligning with greater adherence to key recommendations of the 2015–2020 Dietary Guidelines for Americans^(5)^ *.* Total HEI-2015 scores were derived from the sum of thirteen component scores and were based on procedures established and documented by the National Cancer Institute^(21)^. The thirteen components were: total fruits; whole fruits (e.g. melons); total vegetables; dark green vegetables and legumes; whole grains; total dairy, total protein foods (e.g. meat, eggs, nuts); seafood and plant proteins; refined grains; added sugars; fatty acids (total monounsaturated plus total polyunsaturated/total saturated); sodium; and total saturated fats. Our analyses were based on the most recent version of the HEI, the HEI-2015^(22)^.

### Demographic variables

Mothers, teenagers and children (except for proxy interviews for 5-year-old children) were asked if they were Hispanic, Latina or of Spanish origin. Respondents were asked to identify their race with the option of choosing from one or more of the following categories: ‘American Indian or Alaska Native’, ‘Asian’, ‘Black or African American’, ‘Native Hawaiian or other Pacific Islander’, ‘White’ or ‘Other’. Mothers’ age was ascertained by asking, ‘What is your age?’ Education was assessed by asking mothers the question, ‘What is the highest level of school you have completed or the highest degree you have received?’ Response options were ‘8th grade or less’, ‘9th to 12th grade (no diploma)’, ‘high-school graduate or GED completed’ (where GED is General Educational Development), ‘some vocational, trade or business school but no diploma’, ‘completed a vocational, trade or business school programme’, ‘some college credit but no degree (including Associate’s degree)’, ‘college graduate 4-year degree’ or ‘postgraduate or professional degree’. Each mother’s interview concluded with asking a series of questions to identify the number of minors under her care and, when more than one, to randomly select one child or teenager. The gender and age of the selected child or teenager were recorded during this process and, for teenagers only, confirmed during the subsequent interview.

### Data matching, cleaning and conversion

ASA24 responses were merged with the demographic variables using a unique identification number appearing in both data sets. Data cleaning involved implementing corrections for selected dietary responses based on the criteria established by the National Cancer Institute^(23)^. Due to known ASA24 database errors, corrections to the data for each version of the ASA24 used during the time period of this study (the ASA24-2014 in 2015 and 2016 and ASA24-2016 in 2017) involved adjusting the nutritional values for reported foods and retotalling computed variables to include the adjusted values on a case-by-case basis. The ASA24-2014 used the MyPyramid Equivalents Database (MPED) for determining total nutrition and supplemental values, whereas the ASA24-2016 relied on the USDA’s Food Patterns Equivalents Database (FPED). A SAS macro supplied by the National Cancer Institute^(24)^ was employed to bring the ASA24-2014 data into alignment with the ASA24-2016 data, which was critical for computing and comparing the scores obtained in 2015 and 2016.

### The analytic sample

Partial interviews, defined as incomplete ASA24 interviews, and those with implausible kilojoule intake were excluded from the analyses. The National Cancer Institute has published cut-off points for nutrient outlier data for adults. Total energy intake during a 24 h period for adult women outside the range of 2510·4 to 18 409·6 kJ was considered suspect^(23)^ and omitted from our analyses. The criterion for outliers for teenagers and children was <2719·6 or >24 267·2 kJ.

### Statistical analyses

Frequencies were conducted to describe the demographic characteristics of the mothers, teenagers and children. Multiple linear regression models were developed to examine changes over time for the five continuous outcome variables for each age group. Significant (*α* ≤ 0·05) changes over time were assessed by creating the independent variable for the present study representing 3-year trends, coded as 0 for 2015, 1 for 2016 and 2 for 2017. For the linear regression analyses, the adjusted means for the outcome variables by year and the beta coefficients and their confidence intervals for the trend variable are presented.

Our study objectives are interpreted based on the trend findings from the linear regression analyses for the complete 3-year sample (seventeen counties in 2015 and thirteen additional counties in 2016 and 2017). To assess whether observed trends from the complete sample were an artifact of including responses from individuals from the additional counties, we replicated the analyses and also present the regression coefficients for 3-year trends for interviews completed from only those mothers, teenagers and children from the original seventeen counties.

Race/ethnicity and age in years were covariates in all regression analyses. Race/ethnicity was effect coded and entered into the regression models as Latino (‘Hispanic, Latina or of Spanish origin’ and no racial group identified), African Americans and Whites, with responses to other ethnic categories and missing data (Other/Missing) serving as the reference group. Age was centred on the mean values for each age group.

The regression models for mothers included levels of education. The reference category for education was high-school graduate, GED and vocational schooling (coded as –1). Mothers with less than a 12th grade education and no diploma were coded as 1 *v*. 0, and mothers with highest level of education including some college to a postgraduate or professional degree were coded 1 *v*. 0. The analyses for teenager and child respondents included gender (females = 1) as a covariate.

Data merging, cleaning and coding were conducted with SAS version 9.4. IBM SPSS Statistics version 25.0 was used for the frequencies and regression analyses.

## Results

Sample sizes and the number of interviews completed differed across the 3 years due to budget limitations and delayed contractual arrangements (Table 1). Over the 3-year period, 14 005 mothers were successfully recruited for survey participation. Response rates for the 2015, 2016 and 2017 surveys were 46·6, 41·4 and 40·9 %, respectively (American Association for Public Opinion Research RR4)^(25)^. Response rates for teenagers and children ranged from 64·3 to 73·9 % with denominators based on the number of total mothers interviewed from sampled mother/teenager and mother/child households. Interviews were conducted with 3441 teenagers and 6224 children over the 3-year period. Partial interviews represented less than 5 % (range = 0·32–4·3 %) of total interviews across age groups and survey years. Interviews discarded from the analyses due to implausible kilojoule intake ranged from 1·5 to 5·1 % of interviews within age groups and survey years. Overall, 212 mothers, twenty-six teenagers and sixty-four children were excluded from analyses due to issues affecting data quality. The remaining interviews were considered to consist of valid dietary responses and totalled 13 247 for mothers, 3293 for teenagers and 6043 for children, for an overall sample of 22 583.

**Table 1:** Sample sizes, response rates and processes for developing the analytic data set for dietary analyses of mothers, teenagers and children from low-income households, California Family Health Study, 2015 through 2017

	Mothers, ≥18 years			Teenagers, 12–17 years			Children, 5–11 years		
	2015	2016	2017	2015	2016	2017	2015	2016	2017
Sampled	4332	16 904	12 335	754	2547	1932	1170	4373	3050
Total interviews	1988	7000	5017	542	1638	1261	803	3167	2254
Response rate (%)†	46·6	41·4	40·9	71·9	64·3	65·3	68·6	72·4	73·9
Partial ASA24 interviews	86	119	7	13	9	4	23	30	11
% of total interviews	4·33	1·70	0·14	2·40	0·55	0·32	2·86	0·95	0·49
Complete interviews (total ASA24 interviews – partial ASA24 interviews)	1902	6881	5010	529	1629	1257	780	3137	2243
Implausible kJ‡	97	280	169	25	52	45	30	53	34
% of complete interviews	5·1	4·1	3·4	4·7	3·2	3·6	3·8	1·7	1·5
Valid dietary responses (completed interviews – implausible kJ)	1805	6601	4841	504	1577	1212	750	3084	2209

ASA24, Automated Self-Administered 24-hour Dietary Assessment Tool.

† Calculated based on disposition code 2.2 per the definitions of outcome rates for surveys, American Association for Public Opinion Research^(25)^.

‡ For mothers, <2510·4 or >18 409·6 kJ; for teenagers and children, <2719·6 or >24 267·2 kJ.

Most of the interviewed mothers, teenagers and children from SNAP-Ed eligible households were Latino (Table 2). The mean age of mothers was 38·7 years. Teenagers and children were 14·9 and 8·3 years old on average, and half of them were females. About three in ten (29·8 %) mothers reported less than a high-school education. The highest level of education was high-school graduate for 22·7 % of the sample and some college for 26·4 % of participating mothers.

**Table 2:** Demographic characteristics of mothers, teenagers and children with valid dietary interviews, California Family Health Study, 2015 through 2017

	Mothers, ≥18 years (*n* 13 793)†	Teenagers, 12–17 years (*n* 3415)†	Children, 5–11 years (*n* 6160)†
Race/ethnicity (%)			
Latino	64·3	67·2	68·6
White	16·5	13·3	16·7
African American	12·9	15·8	13·7
Other	4·1	2·7	2·9
Missing	2·1	0·9	1·1
Age (years)			
Mean	38·7	14·9	8·3
Median	38·0	14·9	8·1
Gender (%)			
Female		49·4	49·6
Male		48·7	49·6
Missing		1·8	0·7
Education (%)			
8th grade or less	14·9		
9th to 12th grade	14·9		
High-school graduate	22·7		
Some vocational	1·3		
Completed vocational programme	6·6		
Some college	26·4		
College degree	9·3		
Postgraduate or professional degree	2·1		
Missing	1·8		

† Based on complete interviews.

### Intake of fruits and vegetables with 100 % fruit juice

Among mothers, the adjusted means for consumption of fruits and vegetables with 100 % fruit juice were 3·14 cups/d in 2015 and 3·44 cups/d in 2017, for an increase of 0·3 cups/d on average (Table 3). Teenagers’ diets included 0·4 more daily cups of fruits and vegetables with 100 % fruit juice in 2017 than in 2015. Over the 3-year period, children’s intake of fruits and vegetables with 100 % fruit juice was 2·81 cups/d in 2015, 3·05 cups/d in 2016 and 3·16 cups/d in 2017. The regression coefficient for the 3-year trend variable was significant for mothers (*B* = 0·09; 95 % CI 0·02, 0·16), teenagers (*B* = 0·17; 95 % CI 0·02, 0·32) and children (*B* = 0·16; 95 % CI 0·06, 0·26). For these and the remainder of the findings presented in Table 3, the regression coefficients for the 3-year trend variable were equivalent, in terms of statistical significance, for the complete sample and those models based on the original seventeen-county sample.

**Table 3:** Trends in dietary behaviours for mothers, teenagers and children from low-income households, California Family Health Study, 2015 to 2017

	Mothers, ≥18 years		Teenagers, 12–17 years		Children, 5–11 years	
	Adjusted estimate†	95 % CI	Adjusted estimate†	95 % CI	Adjusted estimate†	95 % CI
Fruits and vegetables with 100 % fruit juice, mean cups/d						
2015	3·14	2·99, 3·28	2·87	2·58, 3·16	2·81	2·60, 3·01
2016	3·53	3·29, 4·06	3·20	3·18, 3·83	3·05	3·02, 3·50
2017	3·44	3·29, 3·89	3·27	3·19, 3·98	3·16	3·03, 3·74
*B* for linear trend across years						
Complete sample‡	0·09*	0·02, 0·16	0·17*	0·02, 0·32	0·16**	0·06, 0·26
Original seventeen counties§	0·11**	0·04, 0·19	0·20**	0·05, 0·36	0·18***	0·07, 0·28
Fruits and vegetables without 100 % fruit juice, mean cups/d						
2015	2·82	2·69, 2·94	2·43	2·17, 2·68	2·40	2·22, 2·58
2016	3·23	2·95, 3·77	2·79	2·69, 3·41	2·69	2·59, 3·19
2017	3·12	2·95, 3·55	2·81	2·70, 3·47	2·72	2·60, 3·23
*B* for linear trend across years						
Complete sample‡	0·08**	0·02, 0·15	0·15*	0·02, 0·28	0·12**	0·03, 0·21
Original seventeen counties§	0·11**	0·04, 0·17	0·19**	0·05, 0·32	0·14**	0·04, 0·23
Sugar-sweetened beverages, mean cups/d						
2015	1·12	1·04, 1·20	1·15	0·98, 1·31	0·78	0·70, 0·86
2016	0·99	0·79, 0·95	1·23	1·14, 1·48	0·71	0·56, 0·73
2017	1·05	0·89, 1·06	1·17	1·02, 1·38	0·72	0·57, 0·74
*B* for linear trend across years						
Complete sample‡	−0·01	−0·05, 0·03	−0·003	−0·09, 0·08	−0·02	−0·06, 0·02
Original seventeen counties§	−0·02	−0·06, 0·02	−0·008	−0·10, 0·08	−0·02	−0·07, 0·02
Water, mean cups/d						
2015	6·76	6·48, 7·05	4·50	3·99, 5·01	3·26	2·96, 3·55
2016	7·44	7·06, 8·42	5·70	5·03, 7·42	3·86	3·56, 4·78
2017	8·05	7·07, 9·64	6·70	5·05, 9·45	4·48	3·57, 6·03
*B* for linear trend across years						
Complete sample‡	0·63***	0·49, 0·78	1·08***	0·82, 1·34	0·62***	0·47, 0·76
Original seventeen counties§	0·65***	0·50, 0·80	1·12***	0·85, 1·39	0·62***	0·47, 0·78
Healthy Eating Index-2015, mean score						
2015	53·3	52·6, 54·0	49·6	48·2, 50·9	51·8	50·8, 52·8
2016	55·8	54·0, 59·1	51·4	50·9, 54·6	53·4	52·9, 56·1
2017	56·1	54·1, 59·7	51·7	51·0, 55·3	54·4	52·9, 58·1
*B* for linear trend across years						
Complete sample‡	1·11***	0·76, 1·46	0·92**	0·24, 1·60	1·22***	0·71, 1·73
Original seventeen counties§	1·19***	0·82, 1·55	0·80*	0·10, 1·49	1·33***	0·80, 1·85

*B*, unstandardized regression coefficient.

*P < 0·05, **P < 0·01, ***P < 0·001 for trend in outcome.

† Adjusted for race/ethnicity, age and highest level of education for mothers; and race/ethnicity, age and gender for teenagers and children.

‡ Analyses based on households sampled from seventeen counties in 2015 and thirteen additional counties in 2016 and 2017.

§ Three-year analyses based on original seventeen counties sampled in 2015. Findings presented to examine whether linear trends are an artifact of additional counties sampled in 2016 and 2017.

### Intake of fruits and vegetables without 100 % fruit juice

Intake of fruits and vegetables without 100 % fruit juice also increased between 2015 and 2017 for mothers (from 2·82 to 3·12 cups/d), teenagers (from 2·43 to 2·81 cups/d) and children (from 2·40 to 2·72 cups/d). The increase for fruits and vegetables without 100 % fruit juice equated to 0·30 cups/d for mothers (*B* = 0·08; 95 % CI 0·02, 0·15), 0·38 cups/d for teenagers (*B* = 0·15; 95 % CI 0·02, 0·28) and 0·32 cups/d for children (*B* = 0·12; 95 % CI 0·03, 0·21). Subtracting the adjusted mean for fruits and vegetables with from that without 100 % fruit juice reveals that almost half a cup of 100 % fruit juice was consumed by teenagers (0·46 cup) and children (0·45 cup) daily in 2017, and mothers’ daily consumption of 100 % fruit juice averaged 0·32 cups in 2017.

### Consumption of sugar-sweetened beverages

In 2015, mothers drank a mean of 1·12 cups of SSB daily, teenagers and children reported consuming 1·15 and 0·78 cups of SSB daily, respectively. Per-year mean intake of SSB for mothers, teenagers and children remained stable (*P* ≥ 0·05) over the 3-year period.

### Consumption of water

Mothers and children reported drinking more than one additional cup of water per day in 2017 than 2015, representing significant increases (*B* = 0·63; 95 % CI 0·49, 0·78 for mothers; *B* = 0·62; 95 % CI 0·47, 0·76 for children). Daily water intake increased by two cups for teenagers (*B* = 1·08; 95 % CI 0·82, 1·34). Daily water intake by 2017 was 8·05 cups/d among mothers, 6·70 cups/d among teenagers and 4·48 cups/d among children.

### Diet quality

Mean HEI-2015 scores increased in a linear fashion and in directions indicative of healthier food and beverage intakes across all three age groups. By 2017, the mean HEI-2015 score for mothers was 56·1; for teenagers, 51·7; and for children, 54·4. The linear increases for mothers (*B* = 1·11; 95 % CI 0·76, 1·46), teenagers (*B* = 0·92; 95 % CI 0·24, 1·60) and children (*B* = 1·22; 95 % CI 0·71, 1·73) were significant.

## Discussion

The objectives of the present study were to examine dietary behaviours and diet quality, over a 3-year period, among mothers, teenagers and children from randomly sampled SNAP-Ed eligible households in California. The five outcome variables examined in the study were selected because they: (i) have been identified in the field of nutrition as main indicators of healthy dietary behaviours; (ii) align with California SNAP-Ed programme goals and objectives; (iii) are of value in informing public health nutrition policy and programme decision making; and (iv) can be used to assess trends in future negative health outcomes and related costs associated with poor dietary behaviours.

Our analyses revealed significant increases in the intake of fruits and vegetables (with or without 100 % fruit juice) among mothers, teenagers and children. We found increases among mothers and children of 0·3 cups/d, on average. This magnitude of change corresponds to the criterion used by the USDA to consider an intervention to be effective^(26)^. The increase in quantity of fruits and vegetables among teenagers of one-third of a cup daily exceeds the USDA criterion.

Findings from the California Family Health Study for fruit and vegetable intake are most comparable to studies using NHANES data; both studies involve population-based surveys and administration of a 24 h dietary recall assessment. Accordingly, we calculated the proportion of mothers participating in the California Family Health Study in 2017 who met or exceeded the MyPlate recommendation of 2·0 cups of fruit daily^(27)^. We found that 28·6 % of SNAP-Ed eligible mothers met this recommendation in 2017 compared with 21·6 % of adults in the USA participating in the 2007 through 2012 NHANES surveys^(28)^. Overall, our findings suggest that public health efforts promoting fruit and vegetable intake to low-income Californians may be producing their intended effects, and low-income SNAP-Ed eligible mothers in California have reached fruit and vegetable consumption levels equivalent to national averages representing all income levels.

We found that intake of SSB remained unchanged over the 3-year study period, but daily water consumption increased significantly by more than one cup for mothers and children and two cups for teenagers. In California, SSB-reduction strategies are predominantly delivered through the California Department of Public Health’s ‘Rethink Your Drink’ initiative, which is designed to provide multipronged messages to promote healthful beverage intake, such as noting the elevated levels of sugar in SSB, the health consequences of consuming so much sugar and the healthful alternative to SSB, specifically water^(29)^. If indeed such interventions have produced behaviour change, it appears that they have been effective in increasing water intake, but not as a replacement for levels of SSB consumption.

From a public health perspective our most important findings were that overall diet quality improved from 2015 to 2017 for mothers, teenagers and children. HEI-2015 scores were calculated based on established procedures and included the intake of vegetables and fruits and the consumption of other healthful foods such as whole grains and protein foods, as well as unhealthful foods such as refined grains, saturated fats and added sugars. These findings are in line with the preponderance of messages communicated through California SNAP-Ed interventions. In fiscal year 2018, the majority (68 %) of messages included in the 61 549 reported California SNAP-Ed interventions promoted eating fruits and vegetables; 56 % included the USDA MyPlate recommendations, such as ‘make half your plate fruits and vegetables’ and ‘drink and eat beverages and food with less sodium, saturated fat, and added sugars’^(27)^.

The findings related to increased fruit and vegetable consumption, complemented by improved diet quality scores, suggest that low-income Californians are changing their dietary behaviours for the better. Moreover, greater proportions of lower-income Californians may have benefited in 2017, compared with 2015, from a decreased risk of cancer, CVD and type 2 diabetes, those diseases shown to decline with increased fruit and vegetable consumption^(2–4)^.

A major limitation of our study is that we cannot ascertain the degree to which SNAP-Ed interventions, and changes in the types of programming across years, may have been responsible for the encouraging 3-year trends. The observed improvements in healthier dietary behaviours do correspond with the growth in the number of California SNAP-Ed sites that adopted policy, system and environmental change approaches, from 682 in 2015, to 902 in 2016, to 1180 in 2017 (L Whetstone, unpublished results). More than 2 million Californians were estimated to have been exposed to direct education activities supplemented by policy, system and environmental change approaches in 2017 (L Whetstone, unpublished results). As previously noted, the majority of messages in these direct education activities focused on increasing fruit and vegetable consumption and encouraging adherence to the USDA’s MyPlate recommendations. Future studies should more directly assess whether the types and intensities of SNAP-Ed interventions are related to the unhealthful and healthful dietary outcomes investigated in the present study.

Another major limitation to the California Family Health Study is that response rates never reached 50 % and in fact declined over the 3-year period, from 46·6 to 40·9 %. Over 58 % of non-response can be attributed to non-contact with sampled households (American Association for Public Opinion Research disposition code 2.2)^(25)^. The MEDS database is a state-wide repository for health and public assistance programmes in California, including Medi-Cal, which for various reasons includes a substantial proportion of records with incomplete and erroneous contact information. The lag between appearing in the MEDS database, sampling for the present study and attempted contact with selected households could have exceeded 15 months and unfortunately resulted in sending recruitment letters to old addresses and calling outdated telephone numbers. Nevertheless, the non-response rate was quite high and the degree to which our data were subject to non-response biases is unknown.

Unfortunately, only limited and dated findings are available to compare our diet quality results with all-income populations across the USA. Recall was based on the previous 24 h, which may represent all respondents’ usual dietary behaviours. Finally, while our findings may be generalized to mothers, teenagers and children from SNAP-Ed eligible households, the degree to which they are applicable to other low-income, at-risk populations within and outside California, as well as in countries other than the USA, is unknown.

The strengths of our study include probability-based sampling of SNAP-Ed-eligible households, use of a validated 24 h dietary recall methodology, administration of the ASA24 by well-trained interviewers, and the consistency in recruitment and interview procedures during the 3-year period. The observed significant 3-year trends for the subset of California counties originally sampled in 2015 and the complete thirty-county sample suggest that our findings are robust and are not due to the decision to expand the samples of SNAP-Ed eligible households by thirteen counties in 2016 and 2017. Our ability to employ a validated methodology to assess overall diet quality, and the consistency of findings across age groups, represent a major strength of our study and strongly suggest that low-income Californians are participating in more healthful dietary behaviours.

In summary, we observed significant and encouraging improvements in fruit and vegetable consumption and water intake, as well as overall diet quality, among individuals from a low-income, at-risk population. However, we found that mothers, teenagers and children continue to consume added sugars through SSB. Given that increased SSB consumption has been linked to the risk of type 2 diabetes and CVD^(8)^, and with metabolic syndrome among adults^(7)^ and adolescents^(6)^, different and more intense SSB-reduction intervention strategies directed at those living in SNAP-Ed eligible households are warranted. The California Department of Social Services, which oversees the State’s SNAP-Ed programme, is currently considering placing greater emphasis on SSB-reduction interventions based on these findings. While increases in intake of fruit and vegetables may lead to substantial public health benefits by lowering the risk of the most common, costly and preventable diseases, observable declines in negative health outcomes and costs due to dietary behaviours among low-income populations in and outside California may not be realized until substantial reductions occur in levels of SSB consumption.

Population-based surveillance efforts using 24 h dietary recall methodologies are costly and resource intense; but, as demonstrated, they have the promise to be worthy investments offering trends and prevalence data for public health nutrition policy and programme decision makers for the justification and (re)allocation of resources and the identification of which types of dietary behaviours are in most need of intervention strategies. They can also be used to assess, in a broad sense, potential trends within at-risk populations of those preventable diseases related to healthful and unhealthful dietary behaviours.
